# Oscillatory interactions between sensorimotor cortex and the periphery

**DOI:** 10.1016/j.conb.2008.01.007

**Published:** 2007-12

**Authors:** Stuart N Baker

**Affiliations:** Institute of Neuroscience, Newcastle University, Henry Wellcome Building, Medical School, Framlington Place, Newcastle upon Tyne, NE2 4HH, UK

## Abstract

Field potential recordings from motor cortex show oscillations in the beta-band (∼20 Hz), which are coherent with similar oscillations in the activity of contralateral contracting muscles. Recent findings have revised concepts of how this activity might be generated in the cortex, suggesting it could achieve useful computation. Other evidence shows that these oscillations engage not just motor structures, but also return from muscle to the central nervous system via feedback afferent pathways. Somatosensory cortex has strong beta-band oscillations, which are synchronised with those in motor cortex, allowing oscillatory sensory reafference to be interpreted in the context of the oscillatory motor command which produced it.

## Introduction

**Figure 1:**
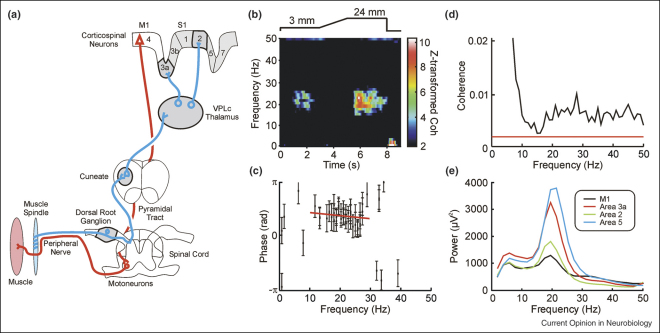
**(a)** Example descending (red) and ascending (blue) pathways which could mediate corticomuscular coherence. **(b)** Task-dependence of corticomuscular coherence. Coherence is shown as a function of frequency (*y*-axis) and time during task performance (*x*-axis), whilst a human subject moved the levers of a precision grip manipulandum according to the displacement target shown schematically above the colour map. Coherence only appears during steady holding phases, and is larger following large movements than small ones. The colour scale has been thresholded so that non-significant coherence appears black. **(c)** The phase of coherence between EEG from sensorimotor cortex and hand muscle EMG in a human subject during steady contraction. Phase is only plotted for frequencies with significant corticomuscular coherence. The red line shows the best-fit straight line to frequencies around the beta-band; the slope of this line was not significantly different from zero. **(d)** Average coherence between forearm EMG and the discharge of seven single afferent units recorded in an awake behaving monkey. Units were putatively identified as muscle spindle primary afferents. Coherence in the beta-band was above significance (red line). **(e)** Comparison of the power of beta-band oscillations in local field potential recorded from different monkey cortical areas. Although oscillations can be seen in all areas illustrated, they are stronger in S1 (area 3a and 2) and posterior parietal cortex (area 5) than in M1 (area 4). (b) redrawn from [1^•^]; (c) redrawn from [21^••^]; (d) redrawn from [24^••^]; (e) redrawn from [13^••^].

Field potential recordings from motor cortex show oscillatory activity. The exact frequency varies between individuals, but power-spectral peaks in both the ‘alpha’ and ‘beta’ bands (∼10 Hz, and 15–30 Hz) are commonly seen [1^•^]. The cortical activity around 20 Hz is coherent with similar oscillations in the electromyogram (EMG) of contralateral contracting muscles; by contrast, corticomuscular coherence is usually absent for the 10 Hz band [2]. Oscillations and corticomuscular coherence are abolished during movement (see Figure 1b), and appear most strongly during rest or periods of steady contraction following a movement [3]. In the visual system, higher frequency oscillations (∼40 Hz) have been intensively investigated; this work has spawned detailed theories of their function, which make experimentally testable predictions [4–6]. Although beta-band oscillations in motor cortex have also been the subject of much experimental investigation, at present we still lack mechanistic functional models. This review focuses mainly on work published in the past two years, and uses recent experimental findings to suggest a possible functional role for this activity.

## Cortical generation of oscillations

Previous experimental and modelling work has elucidated the way in which local cortical circuits can generate stable network oscillations [7]. Inhibitory interneurones are critical to this process: recurrent excitation leads to a crescendo of activity, which is damped down by delayed but powerful inhibition. Oscillation frequency is altered by the time course of inhibition [8]. Stable oscillations can be observed even in networks without excitatory neurones. Until recently, this mechanism — largely worked out for gamma-band oscillations in hippocampal cortex — was assumed to underlie the slower beta-band activity of the motor system. It undoubtedly plays a role: pharmacologically enhancing cortical inhibition increases the size of beta-band power spectral peaks [9]. However, a recent report [10^••^] demonstrated that a quite separate mechanism is also present in sensorimotor cortex. Layer V pyramidal neurones have gap junctional connections between their axons, leading to strong electrical coupling. This can produce stable population oscillations even when synaptic potentials are pharmacologically blocked. In the slices of rat somatosensory cortex used in this study, inhibitory interneurones appeared to generate a gamma rhythm in the superficial cortical layers, at the same time as a beta-band oscillation in layer V produced by gap-junctional interactions. It remains to be seen whether such a clear separation of frequency by layer occurs in the intact, awake animal.

A further contributor to oscillatory activity has also recently been identified. Neurones in the motor cortex can exhibit an intrinsic tendency to rhythmic firing [11,12]. Direct evidence from intracellular recordings, as well as indirect arguments based on statistical analysis of extracellular spikes, suggest that this is produced by the shape of the after-hyperpolarisation trajectory. Following a spike, the membrane potential shows a clear peak, which tends to induce repetitive firing at rates close to beta-band frequencies. A study last year compared oscillatory activity in motor and somatosensory cortex [13^••^]. Both pre-central and post-central cortex showed oscillations in local field potential recordings; in fact, oscillations were stronger in S1 than in M1 (Figure 1e). However, the intrinsic tendency to rhythmic firing was most pronounced for identified corticospinal neurones in M1. This tells us firstly that peaked post-spike membrane trajectories cannot be necessary for rhythmogenesis: somatosensory areas manage to produce robust beta-band oscillations even though most cells have a monotonically rising, rather than peaked, post-spike trajectory.

Secondly, earlier work showed that the spike train of a simple integrate-and-fire neuron represents oscillations in its input rather poorly [2]. By contrast, peaked post-spike membrane potential trajectories will enhance the ability of a cell to lock its discharge to oscillatory input. The specific association of this property with corticospinal output neurones in M1 may imply that normal function requires oscillations to reach the spinal cord, and that the system has accordingly evolved to maximise the fidelity of oscillatory transmission in the corticospinal tract.

It is easy to assume that motor cortical oscillations are a global phenomenon, synchronously engaging all active cells. This concept underlies previous suggestions that motor cortical oscillations represent an ‘idling rhythm’ [14]: something for cells to do when they are not busy controlling movements. However, simultaneous recordings of local field potential from many spatially separated sites in awake behaving monkeys reveal a more complex story. Rather than being uniformly synchronised across locations, activity can organise into travelling waves [15^••^]. The direction of wave travel tends to align along a major axis (anterior–posterior in primary motor cortex; medio-lateral in dorsal pre-motor cortex), and the waves encode information about the cues guiding behaviour in both their amplitude and phase. This observation marks an important advance. Travelling waves have been reported in a wide range of sensory systems previously, and theoretical considerations suggest they could perform useful computation [16]. The extension of these ideas to the motor system provides new vistas for how oscillatory activity might generate useful processing.

## Oscillatory coupling between cortex and periphery

Previous work on corticomuscular coherence has assumed that the phenomenon results from cortically generated oscillations ‘spilling over’ into the corticospinal tract, and thence necessarily influencing motoneurons and muscle. It is certainly the case that the corticospinal output neurones of motor cortex are intimately associated with the circuits responsible for oscillations [17]. It has been directly shown that cortical oscillations are effectively transmitted by a population of corticospinal axons [2].

However, several earlier papers hinted that the situation was not quite so straightforward. Administration of benzodiazepines markedly increases the power of cortical oscillations, but leaves corticomuscular coherence unchanged. This cannot be explained if activity simply propagates from cortex to muscle [9]. Secondly, transmission of activity from cortex to muscle is associated with a neural conduction delay, comprising central and peripheral axonal conduction times plus the synaptic delay at the motoneurone. When we calculate corticomuscular coherence for such a system, the coherence phase should be linearly related to frequency, with a slope equal to 2*π* radians times the delay. Some earlier reports claim to find this relationship [18,19]; others do not [20].

A detailed re-examination of the phase issue was carried out by Riddle and Baker [21^••^]. In the normal human subjects examined, around half showed a linear phase–frequency relationship. However, the slope of this relationship implied a delay (to hand muscles) of ∼10 ms. This is less than half the minimum conduction time from cortex to the hand over fast corticospinal pathways. In the rest of the subjects, the phase difference between cortex and muscle appeared constant over a wide range of frequencies (see example in Figure 1c). This study then went a stage further, and perturbed conduction delays by cooling the arm. After cooling the skin at 10 °C for 112−2 h, the peripheral motor conduction time from spinal cord to hand muscles was increased by up to 70%. This was estimated using electrical stimulation of the peripheral nerve — an objective and reliable standard approach unrelated to spontaneous oscillations. In subjects where coherence phase originally appeared constant at different frequencies, cooling appeared to displace phase upwards, but without introducing a frequency dependence. In subjects with linear phase–frequency dependence, the slope of the relationship did increase. However, the available data suggested that the size of this increase was around twice the known increase in motor conduction time.

None of these findings can be reconciled with the idea that corticomuscular coherence arises solely from cortical output pathways. An obvious additional route which we might consider is feedback from the periphery (see illustration of possible pathways in Figure 1a). It is well known that motor as well as somatosensory cortex receives powerful input from receptors in skin, muscle and joints [22]. If both feedback and feedforward pathways are contributing, this could produce the complex and heterogeneous phase–frequency relationships which are seen in experimental data. It has previously been shown by computational modelling, for example, that reciprocally coupled neural networks can synchronise at zero-phase lag [23]. Similar mechanisms might lead to the constant phase synchronisation between cortex and the periphery. In addition, as described above arm cooling produced an increase in the delay estimated from corticomuscular coherence phase which was around twice that expected from the increased motor conduction time [21^••^]. Cooling will alter conduction times in both sensory and motor nerves similarly; the observed changes in phase delay may thus match more changes closely in the total feedback loop delay, rather than just the motor component.

A recent study analysed the discharge of peripheral afferents recorded from the dorsal root ganglia of awake behaving monkeys [24^••^]. Afferent spiking was coherent with oscillations in muscle activity over a wide frequency range — including the beta band. This was also the case for a small number of recordings highly likely to be from Group Ia muscle spindle afferents (Figure 1d); by contrast, afferents suggested to originate from cutaneous receptors did not represent muscle oscillations in their firing. The oscillatory signal does therefore seem to return to the central nervous system from muscles.

Several key structures which receive and process incoming somatosensory information seem to be part of this oscillating network. Neurones in the deep cerebellar nuclei [25], and somatosensory and posterior parietal cortex [26^•^] fire spikes, which are coherent with motor cortical oscillations. In each case, the spikes occur roughly a quarter of an oscillation cycle before the negative peak of local field potential oscillations in M1. This is similar to the spiking behaviour of corticospinal neurones within M1 itself [2], and there are biophysical reasons to believe that this phase difference between the experimentally recorded signals represents zero-phase synchronisation between the underlying neural activities [2].

If oscillations are involved in somatosensory, as well as motor pathways, we would expect disturbed sensation to impact on coherence. This is indeed the case. In patients lacking large fibre afferents, oscillatory coupling between muscles is markedly reduced [27], though cortical oscillatory power in the beta-band is not significantly different from normals [28]. Experimentally induced anaesthesia of the digits also reduces inter-muscular coherence [29].

One recent report appeared to indicate that feedback processes are not involved in corticomuscular coherence [30^•^]. In normal human subjects, motor and somatosensory cortices are adjacent, and it is difficult to resolve their respective contributions to coherence using non-invasive recordings such as electroencephalography or magnetoencephalography (MEG). However, when motor cortex on one side is damaged peri-nataly, control of the contra-lesional hand can be taken over by the intact, ipsilateral motor cortex. In such individuals, somatosensory processing from the impaired hand is carried out by contralateral S1, but motor commands come from ipsilateral M1. Resolving activity from motor and somatosensory cortices is thus straightforward, as they are in opposite hemispheres. These experiments demonstrated clear corticomuscular coherence between MEG recordings over M1 and EMG, but not from S1. However, the results are especially difficult to interpret, as the sensorimotor networks have undergone extensive reorganisation after a lesion. It may be that the key feature of these patients is a disordered ability for communication between S1 and M1. Rather than the usual dense network of cortico-cortical connections, communication must pass over the corpus callosum. Low firing rates in callosal cells compared with other cortical neurones probably severely limit the efficiency of inter-hemispheric versus intra-hemispheric interaction [31]. A preliminary report using invasive recordings from M1 and S1 in normal monkey showed that both areas exhibit corticomuscular coherence (CL Witham and SN Baker, 2007, *Abstract, IBRO Satellite Meeting, Darwin, Australia*).

## Functional role for corticomuscular coherence

The assumption was often made that beta-band oscillations played some role in the control of movement because initial reports observed them in motor cortex. However, these oscillations are suppressed by movement (Figure 1b) — or even by imagining a movement [32] — making it unlikely that they play a crucial role in motor performance. Recent work has made it clear that oscillations are a sensorimotor phenomenon. This opens up new possibilities for their functional role.

One attractive idea is that descending oscillations in the motor command function as a ‘test pulse’ [33]. This known signal is sent by the brain to muscle, and the afferent response is compared to the descending command with the aim of discovering features of the peripheral state. An analogy with radar or sonar systems may be appropriate [24^••^]. In the rat whisker somatosensory system, there is evidence that a comparison of ∼10 Hz oscillatory motor outflow with sensory reafference proceeds via a neural implementation of a phase-locked loop [34,35].

It is interesting that muscle spindle afferents appear to carry oscillatory activity from muscles especially well [24^••^], given the importance of this receptor system for proprioception. Early experiments showed that proprioceptive errors could be produced by muscle vibration at ∼100 Hz, a stimulus which excites spindles especially strongly. However, one previous study tested a range of vibration frequencies [36]. Proprioceptive errors with 20 Hz vibration were in the opposite direction from those produced by higher frequencies. This result might be expected if proprioceptive processing involves a comparison between the expected and actual level of beta-band power returning to the central nervous system via spindle afferents.

If this idea is correct, it suggests that beta-band oscillations could act to ‘recalibrate’ the sensorimotor system following a movement. A study published last year showed that corticomuscular coherence is greater following large movements than after small movements [1^•^; Figure 1b]. Noise in the motor system appears to be ‘signal dependent’: it is not constant, but scales with the size of a movement [37]. If large movements lead to greater subsequent uncertainty in the state of the periphery, this could explain the need for more ‘oscillatory recallibration’, and the observed greater corticomuscular coherence.

Proprioceptive inputs are especially important during the acquisition of novel motor skills. Perez *et al.* [38^•^] trained subjects to perform a complex visuo-motor task involving a novel use of the ankle joint. Following training on this task, corticomuscular coherence was transiently elevated, although it returned to baseline levels on average by 10 min after the end of the training session. The authors interpreted the coherence rise as reflecting increased corticospinal drive to muscles, and this indeed may be part of the explanation. However, any system involved in sensorimotor integration, and the interpretation of proprioceptive information, would also probably be strongly recruited by this task. The elevated coherence might then reflect the continued consolidation of the learned skill in its proprioceptive context.

A quite different view of the functional role of beta-band oscillations has been taken by Brown and coworkers. In several detailed recent studies [39^••^,40,41^•^,42^•^], this laboratory and others have produced strong evidence that beta-band oscillations represent a cortical state which promotes the maintenance of steady motor output. This idea could be reconciled with evidence suggesting a role in sensorimotor recalibration in several ways. It is possible that one of these apparent functions is just an epiphenomenon generated by the action of the other. For example, the presence of beta-band oscillations circulating a sensorimotor loop may create a system which also happens to be especially stable, but is an unintended consequence of the use of oscillations in this way. Initiation of movement would require the disruption of oscillations, and entry into a non-oscillatory mode, which could permit more freedom to represent and process information [43]. Equally, an effective oscillatory stabilisation system might inevitably produce re-afferent oscillations as an unwanted by-product. The latter view cannot explain, however, why oscillations should engage not just M1 but also S1 so effectively.

Alternatively, it is more probably that these two putative functions of beta-band oscillations represent incomplete descriptions of the same process from different perspectives. Oscillations may hold overt motor output constant in order to render the interpretation of the proprioceptive state more effective. Periodic monitoring of the state of the periphery may facilitate rapid feedback corrections to maintain a constant output. The effective fusion of these two overlapping viewpoints into a satisfying unifying hypothesis is a major challenge in the field.

This review has concentrated on beta-band oscillations in the sensorimotor system, which appears mainly during rest or steady contraction. Two recent studies have reported corticomuscular coherence at higher frequency (∼40 Hz, ‘gamma band’). One report shows that gamma-band corticio-muscular coherence appears during a demanding force tracking task [44^•^]. The other shows that coherence at these higher frequencies increases with increasing expectation of the need to move [45^•^]. At this stage, it is not clear whether all corticomuscular coherence is subserving the same function, with the precise frequency merely an artefact of the experimental conditions, or whether oscillations at different frequencies perform distinct functions. Given the emerging evidence that these rhythms may be generated in the cortex by distinct mechanisms [10^••^], it is entirely possible that their functional contributions are equally distinct.

## Conclusions

Earlier work, largely in the visual system, suggested that synchronised oscillations could be important for linking and communicating information between different cortical areas. Recent findings in the motor system have extended this idea to encompass key centres outside the cortex, including spinal cord, muscle, and afferent nerves. Beta-band oscillations may have a role in sensorimotor integration, somehow recalibrating the system following a movement and thus preparing for the next movement. The challenge now is to make some of these ideas more concrete. What information about the periphery could best be learnt by probing with oscillations? How could ascending oscillations be processed by central pathways to yield a representation useful in subsequent motor control? What are the inter-relationships between oscillatory feedback, and the non-oscillatory reafference which occurs during movement itself and which is so critical for successful motor execution? Answering these questions will require careful experimentation, but may finally give us the detailed mechanistic understanding of this activity which has so far proved elusive.

## References and recommended reading

Papers of particular interest, published within the annual period of review, have been highlighted as:

• of special interest
•• of outstanding interest
